# C3/4 Stenosis and Lower Limb Functional Impairment in Cervical Spondylotic Myelopathy: A Retrospective Exploratory Study

**DOI:** 10.7759/cureus.109847

**Published:** 2026-05-28

**Authors:** Keisuke Otsuka, Yosuke Horiuchi, Osahiko Tsuji

**Affiliations:** 1 Department of Orthopedic Surgery, Saiseikai Utsunomiya Hospital, Utsunomiya, JPN; 2 Department of Orthopedic Surgery, Toho University Ohashi Medical Center, Meguro City, JPN

**Keywords:** c3/4 stenosis, cervical spondylotic myelopathy, japanese geriatrics, lower extremity dysfunction, posterior column

## Abstract

Cervical spondylotic myelopathy (CSM) is a common clinical syndrome caused by degenerative changes of the cervical spine and frequently presents with lower extremity dysfunction. However, the relationship between specific cervical levels and lower extremity functional impairment remains unclear. This retrospective single-center study investigated the association between C3/4 stenosis and lower extremity dysfunction in patients with CSM. Twenty-four patients who underwent surgical treatment between April 2024 and June 2025 were included after excluding those with comorbidities affecting lower extremity function. C3/4 stenosis was defined as an anteroposterior-to-transverse diameter ratio of less than 0.4 on axial T2-weighted magnetic resonance imaging (MRI). Clinical severity was evaluated using the Japanese Orthopaedic Association (JOA) score and the Japanese Orthopaedic Association Cervical Myelopathy Evaluation Questionnaire (JOA-CMEQ). Patients with C3/4 stenosis were significantly older and demonstrated significantly worse lower-extremity motor and sensory scores on the JOA scale, as well as poorer lower-extremity function scores on the JOA-CMEQ. After adjustment for age, C3/4 stenosis was associated with lower extremity sensory dysfunction and patient-reported lower extremity disability. These findings suggest that patients with C3/4 stenosis demonstrated worse lower extremity-related functional impairment in this retrospective exploratory cohort, possibly reflecting the anatomical organization of sensory pathways within the posterior column at upper cervical levels. However, the independent contribution of C3/4 pathology could not be definitively determined from the present analysis.

## Introduction

Cervical spondylotic myelopathy (CSM) is a clinical syndrome resulting from degenerative changes of the cervical spine and is widely recognized as a leading cause of nontraumatic spinal cord dysfunction in adults [1]. With population aging, its prevalence has been increasing worldwide. In elderly patients, decreased mobility of the lower cervical spine may lead to compensatory mechanical stress at the upper cervical levels, particularly C3/4 and C4/5, resulting in dynamic compression [2]. Lower extremity dysfunction is one of the most clinically significant manifestations of CSM and is associated with functional decline and prognosis [3].

Previous studies have demonstrated that intramedullary high signal intensity and the degree of spinal cord compression on T2-weighted magnetic resonance imaging (MRI) correlate with lower extremity dysfunction [4]. C3/4 stenosis has been reported to occur more frequently in elderly patients [5]. However, few studies have quantitatively compared lower extremity-related functional outcomes between patients with and without C3/4 stenosis. The Japanese Orthopaedic Association Cervical Myelopathy Evaluation Questionnaire (JOA-CMEQ) enables patient-reported assessment of lower extremity function, but its application in level-specific analyses remains limited [6].

The purpose of this study was to investigate the association between C3/4-level stenosis and lower extremity dysfunction in patients with CSM. We hypothesized that patients with C3/4 stenosis would demonstrate significantly worse lower extremity functional outcomes compared with those without C3/4 involvement.

## Materials and methods

Study design and ethical considerations

This was a retrospective single-center observational study. Patients who underwent surgical treatment for CSM between April 2024 and June 2025 were reviewed. The study was approved by the institutional ethics committee and conducted in accordance with the Declaration of Helsinki. Informed consent was obtained using an opt-out method.

Patient selection

**Figure 1:**
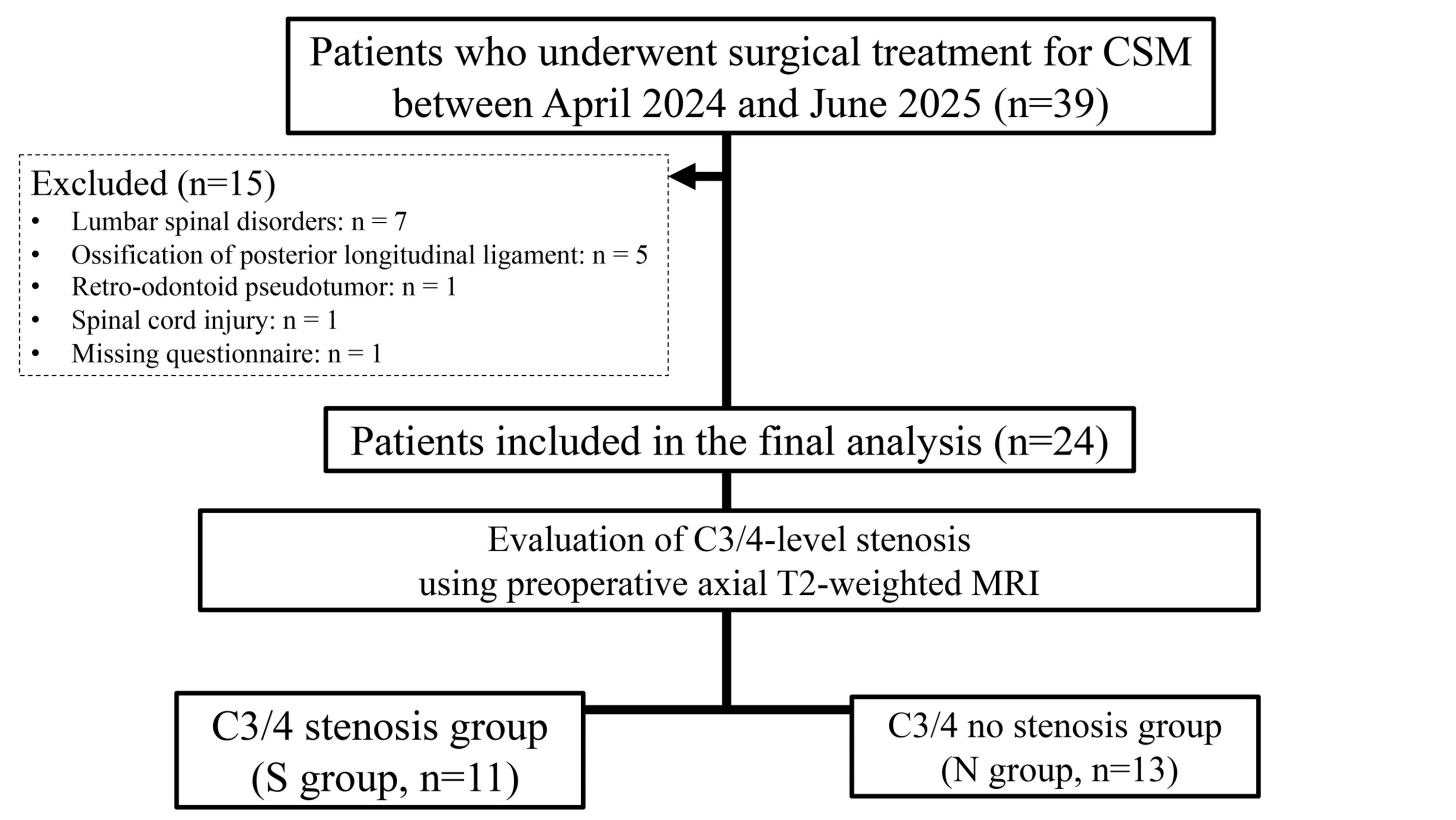
Patient selection flowchart A total of 39 patients who underwent surgical treatment for cervical spondylotic myelopathy between April 2024 and June 2025 were initially identified. After applying exclusion criteria, 24 patients were included in the final analysis and divided into the C3/4 stenosis group (S group, n = 11) and the non-C3/4 stenosis group (N group, n = 13)

A total of 39 consecutive patients who underwent surgery for CSM during the study period were initially identified. Patients were excluded if they had comorbid conditions or other spinal disorders that could affect lower extremity function or confound evaluation of CSM. The reasons for exclusion included concomitant lumbar spinal disorders (n = 7), ossification of the posterior longitudinal ligament (n = 5), retro-odontoid pseudotumor (n = 1), spinal cord injury (n = 1), and missing questionnaire data (n = 1), as summarized in Figure 1. After applying the exclusion criteria, 24 patients were included in the final analysis (Figure 1). Additional information regarding stenosis at other cervical levels, surgical procedures, decompression levels, and the number of decompressed levels was collected and summarized in the supplementary table in the Appendices.

Definition of C3/4 stenosis

C3/4 stenosis was operationally defined using axial T2-weighted MRI measurements, in which the anteroposterior-to-transverse diameter ratio of the spinal cord was calculated, and a value below 0.4 was considered indicative of stenosis based on previously reported criteria [7]. The A/B ratio was selected as a practical and reproducible parameter for group classification in this retrospective clinical study. Image measurements were performed independently by two spine surgeons.

Clinical evaluation

Neurological function was assessed using the JOA score (17-point scale) and JOA-CMEQ. The JOA score includes subscores for upper extremity motor function/sensory function, lower extremity motor function/sensory function, and bladder function [8-10]. The JOA-CMEQ is a validated patient-reported outcome measure that evaluates upper extremity function, lower extremity function, and bladder function [6]. All assessments were conducted preoperatively. The presence or absence of intramedullary T2 high signal intensity on preoperative MRI was additionally evaluated and compared between groups.

Surgical indications

Surgical indications were determined by two board-certified spine surgeons based on detailed neurological examination findings and imaging results. Progressive neurological symptoms, lower extremity dysfunction affecting activities of daily living, and radiographic evidence of spinal cord compression were comprehensively evaluated. Treatment decisions were made according to consistent criteria within the institution. Posterior decompression was primarily performed using skip laminoplasty, a muscle-preserving posterior decompression technique described by Nori et al. [11]. Detailed information regarding surgical procedures, decompression levels, and the number of decompressed levels is provided in the supplementary table in the Appendices.

Statistical analysis

Statistical analyses were performed using EZR (Jichi Medical University, Tochigi, Japan). Continuous variables were compared between groups using Student’s t-test or the Mann-Whitney U test, as appropriate. Categorical variables were compared using the chi-square test. The association between age and clinical outcomes was assessed using Spearman’s rank correlation analysis. To account for the potential confounding effect of age, multivariable linear regression analysis (analysis of covariance) was performed with clinical scores as dependent variables and age and group classification as independent variables. A p-value < 0.05 was considered statistically significant. All analyses were conducted according to predefined statistical procedures.

## Results

Patient characteristics

A total of 24 patients were included in the final analysis, comprising 11 patients in the C3/4 stenosis group (S group) and 13 patients in the non-C3/4 stenosis group (N group). The mean age was significantly higher in the S group than in the N group (76.6 ± 8.2 vs. 56.3 ± 7.6 years; t = -4.03, p < 0.001). There were no significant differences in sex distribution (χ² = 0.035, p = 0.85), body mass index (t = 0.36, p = 0.72), or duration of symptoms (U = 21, p = 0.38) between the two groups (Table 1). Intramedullary T2 high signal intensity was observed in all patients in the S group and in 69% of patients in the N group; however, the difference was not statistically significant (p = 0.14) (Table 1).

**Table 1:** Patient demographics and baseline characteristics BMI: indicates body mass index; SD: standard deviation Comparison of age, sex, BMI, and symptom duration between the C3/4 stenosis group (S group) and the non-C3/4 stenosis group (N group). Data are presented as mean ± SD for continuous variables and number (N, %) for categorical variables. Comparisons between groups were performed using Student’s t-test or the Mann-Whitney U test for continuous variables, and the chi-square test for categorical variables, as appropriate. Test statistics are presented as t values, U values, or χ² values. A p-value < 0.05 was considered statistically significant

Variable	S group (n = 11)	N group (n = 13)	Test statistic	P
Age (years)	76.6 ± 8.2	56.3 ± 7.6	t = -4.03	<0.001*
Sex (male), n (%)	8 (72.7%)	9 (69.2%)	χ² = 0.035	0.85
BMI (kg/m²)	23.6 ± 4.6	25.1 ± 4.3	t = 0.36	0.72
Duration of symptoms (months)	8.7 ± 10.1	8.0 ± 6.3	U = 21	0.38
Intramedullary T2 signal change	11 (100%)	9 (69.2%)	χ² = 2.15	0.14
BMI				

Comparison of JOA scores

The JOA lower extremity motor score was significantly lower in the S group (0.96 ± 0.7) than in the N group (2.31 ± 0.8) (p < 0.01). The JOA lower extremity sensory score was also significantly lower in the S group (1.32 ± 0.5) compared to the N group (1.85 ± 0.4) (p = 0.01). Bladder function scores were significantly lower in the S group (1.64 ± 0.8) than in the N group (2.23 ± 0.4) (p = 0.03). The total JOA score was significantly lower in the S group (7.91 ± 2.4) compared to the N group (11.3 ± 1.5) (p < 0.01). No significant differences were observed in upper extremity motor function, upper extremity sensory function, or trunk sensory function between the two groups (Table 2).

**Table 2:** Comparison of JOA scores between groups JOA: Japanese Orthopaedic Association; VAS: visual analog scale; SD: standard deviation Comparison of motor, sensory, bladder, and total JOA scores between the S group and the N group. Data are presented as mean ± SD. P-values were calculated using Student’s t-test. Test statistics are presented as t values. A p-value < 0.05 was considered statistically significant

Cervical JOA	S group (n = 11)	N group (n = 13)	Test statistic	P
Motor function-hands and fingers	1.55 ± 0.9	2.0 ± 0.6	t = 1.65	0.11
Motor function-shoulder and elbow joints	-0.14 ± 0.3	0 ± 0	t = 1.53	0.14
Motor function-lower extremities	0.96 ± 0.7	2.31 ± 0.8	t = 4.22	＜0.01*
Sensory function-upper extremities	0.96 ± 0.14	1 ± 0	t = 1.09	0.29
Sensory function-trunk	1.64 ± 0.5	1.85 ± 0.4	t = 1.17	0.26
Sensory function-lower extremities	1.32 ± 0.5	1.85 ± 0.4	t = 2.75	0.01*
Bladder function	1.64 ± 0.8	2.23 ± 0.4	t = 2.29	0.03*
Total score	7.91 ± 2.4	11.3 ± 1.5	t = 4.06	<0.01*
VAS for pain or stiffness in neck or shoulders	5.5 ± 3.1	5.6 ± 3.0	t = 0.12	0.90
VAS for tightness in chest	0.46 ± 0.9	0.54 ± 1.4	t = 0.17	0.87
VAS for pain or numbness in arms or hands	7.7 ± 3.0	7.5 ± 2.4	t = -0.23	0.82
VAS for pain or numbness in chest to toe	5.5 ± 3.9	2.6 ± 3.0	t = -1.93	0.07

Comparison of JOA-CMEQ scores

The JOA-CMEQ lower extremity function score was significantly lower in the S group (28.1 ± 24.0) than in the N group (69.9 ± 20.5) (p < 0.01). Upper extremity function and bladder function scores on the JOA-CMEQ were also lower in the S group; detailed values are presented in Table 3.

**Table 3:** Comparison of JOA-CMEQ scores between groups JOACMEQ: Japanese Orthopaedic Association Cervical Myelopathy Evaluation Questionnaire; QOL: quality of life; SD: standard deviation Comparison of upper extremity function, lower extremity function, and bladder function scores between the two groups. Data are presented as mean ± SD. P-values were calculated using Student’s t-test. Test statistics are presented as t values. A p-value < 0.05 was considered statistically significant

Variable	S group (N = 11)	N group (N = 13)	Test statistic	P
Cervical function	71.8 ± 13.9	73.5 ± 16.7	t = 0.25	0.81
Upper extremity function	56.5 ± 21.7	84.6 ± 13.8	t = 3.70	＜0.01*
Lower extremity function	28.1 ± 24.0	69.9 ± 20.5	t = 4.41	＜0.01*
Bladder function	64.2 ± 18.3	80.8 ± 12.9	t = 2.49	0.02*
QOL	39.3 ± 11.6	47.8 ± 9.7	t = 1.88	0.07

Multivariable linear regression analysis

Spearman’s rank correlation analysis demonstrated significant negative correlations between age and JOA bladder function score (ρ = -0.615, p = 0.001), JOA lower extremity motor function score (ρ = -0.751, p < 0.001), JOA total score (ρ = -0.804, p < 0.001), JOA-CMEQ lower extremity function score (ρ = -0.578, p = 0.003), and JOA-CMEQ upper extremity function score (ρ = -0.495, p = 0.014). In contrast, no significant correlations were observed between age and JOA lower extremity sensory function score (ρ = -0.279, p = 0.187) or JOA-CMEQ bladder function score (ρ = -0.344, p = 0.100) (Table 4).

**Table 4:** Spearman’s rank correlation analysis between age and clinical outcomes JOA: Japanese Orthopaedic Association; JOACMEQ: Japanese Orthopaedic Association Cervical Myelopathy Evaluation Questionnaire Correlation coefficients (ρ) and p-values for the relationship between age and each clinical parameter. Correlation coefficients (ρ) and p-values are shown. A p-value < 0.05 was considered statistically significant

		Spearman's ρ	P
JOA	Motor function-lower extremities	-0.751	<0.001*
	Sensory function-lower extremities	-0.279	0.187
	Bladder function	-0.615	0.001*
	Total score	-0.804	<0.001*
JOACMEQ	Lower extremity function	-0.578	0.003*
	Upper extremity function	-0.495	0.014*
	Bladder function	-0.344	0.1

No significant interaction between age and group was observed (p = 0.329), and regression diagnostics showed no evidence of substantial multicollinearity. In multivariable linear regression analysis adjusted for age, age remained an independent predictor of JOA bladder function score (β = -0.026, p = 0.045), JOA lower extremity motor function score (β = -0.042, p = 0.008), and JOA total score (β = -0.114, p = 0.005), whereas group classification was not significantly associated with these outcomes, although poorer JOA lower extremity motor function scores were observed in the C3/4 stenosis group (β = -0.66, p = 0.087). In contrast, group classification was independently associated with JOA lower extremity sensory function score (β = -0.56, p = 0.042) and JOA-CMEQ lower extremity function score (β = -29.96, p = 0.023), while age showed no significant association with these measures. For JOA-CMEQ bladder and upper extremity function scores, age was not significantly associated with either outcome. Group classification was associated with lower JOA-CMEQ bladder function (β = -15.37, p = 0.102) and upper extremity function (β = -19.94, p = 0.057), although these associations did not reach statistical significance (Table 5).

**Table 5:** Multivariable linear regression analysis (ANCOVA) adjusted for age JOA: Japanese Orthopaedic Association; JOACMEQ: Japanese Orthopaedic Association Cervical Myelopathy Evaluation Questionnaire; ANCOVA: analysis of covariance; CI: confidence interval Regression coefficients (β), 95% confidence intervals, test statistics, and p-values evaluating the independent effects of age and group classification on clinical outcomes. Data are presented as a regression coefficient (β) with 95% CI. A p-value < 0.05 was considered statistically significant

		Age β (95% CI)	Test statistic	P	Group β (95% CI)	Test statistic	P
JOA	Motor function-lower extremities	-0.042 (-0.071 to -0.012)	t = -2.94	0.008*	-0.66 (-1.41 to 0.10)	t = -1.80	0.087
	Sensory function-lower extremities	0.002 (-0.019 to 0.023)	t = 0.20	0.847	-0.56 (-1.10 to -0.02)	t = -2.17	0.042*
	Bladder function	-0.026 (-0.052 to -0.001)	t = -2.13	0.045*	-0.15 (-0.81 to 0.51)	t = -0.48	0.635
	Total score	-0.114 (-0.189 to -0.038)	t = -3.14	0.005*	-1.49 (-3.43 to 0.44)	t = -1.6	0.124
JOACMEQ	Lower extremity function	-0.71 (-1.69 to 0.28)	t = -1.5	0.149	-29.96 (-55.27 to -4.66)	t = -2.46	0.023*
	Upper extremity function	-0.49 (-1.29 to 0.31)	t = -1.27	0.217	-19.94 (-40.56 to 0.68)	t = -2.01	0.057
	Bladder function	-0.07 (-0.80 to 0.66)	t = -0.2	0.841	-15.37 (-34.06 to 3.32)	t = -1.71	0.102

## Discussion

This study demonstrated two principal findings. First, patients with C3/4 stenosis were significantly older than those without C3/4 stenosis. Second, C3/4 stenosis was associated with lower extremity dysfunction, as evidenced by significantly lower JOA lower extremity sensory function score and JOA-CMEQ lower extremity function score after adjustment for age. These findings suggest that objective motor dysfunction is strongly influenced by aging, whereas sensory disturbance and patient-reported lower-extremity disability may more directly reflect the effects of C3/4 stenosis itself.

The association between C3/4 stenosis and advanced age observed in this study is consistent with previous reports. Age-related reduction in lower cervical mobility may increase mechanical stress at upper cervical segments, particularly C3/4, leading to dynamic compression [2]. C3/4 stenosis has been reported to occur more frequently in elderly patients [5]. Furthermore, surgical outcome studies have treated C3/4 myelopathy in elderly patients as a distinct clinical entity [12].

The association between C3/4 stenosis and lower extremity dysfunction may be explained by the anatomical characteristics of the upper cervical spinal cord. The posterior column becomes relatively more prominent at higher cervical levels, and the fasciculus gracilis, which transmits proprioceptive input from the lower extremities, is located dorsally and near the midline, making it susceptible to compression at the C3/4 level [13,14]. One possible explanation is that dysfunction of this pathway may contribute to sensory ataxia and lower extremity dysfunction, even in the absence of marked upper extremity involvement. These findings may partially reflect the anatomical organization of sensory pathways within the posterior column of the upper cervical spinal cord [13-16]. In addition, disruption of propriospinal interneuronal networks may also contribute to impaired locomotor coordination, although direct clinical evidence in compressive myelopathy remains limited [17].

A nationwide survey in Japan reported that most traumatic spinal cord injuries occur in elderly individuals and predominantly involve the cervical spine, often resulting from low-energy falls [18]. Given that patients with C3/4 stenosis were significantly older and exhibited greater lower extremity dysfunction in the present cohort, this subgroup may be at increased risk for traumatic cervical spinal cord injury. Although trauma incidence was not directly evaluated, the level-specific association with lower extremity dysfunction may have implications for risk stratification.

This study has several limitations. It was a retrospective single-center study with a small sample size. In addition, no a priori sample size calculation was performed because of the exploratory retrospective study design. Multiple statistical comparisons were performed without formal correction for multiple testing; therefore, the findings should be interpreted cautiously. Dynamic factors and intramedullary signal changes were not quantitatively evaluated. Formal interobserver reliability analysis for MRI measurements was not performed. Although the frequency of intramedullary T2 high signal intensity did not differ significantly between groups, more detailed radiographic analyses may be necessary to further clarify the relationship between spinal cord pathology and lower extremity dysfunction. The association with traumatic spinal cord injury remains speculative. Additionally, C3/4 stenosis may reflect age-related changes in lower cervical mobility. Although multilevel degenerative changes were present in both groups, the possibility that the observed findings reflect generalized cervical degeneration rather than a purely C3/4-specific effect cannot be completely excluded. Despite these limitations, the present study demonstrated an association between C3/4 stenosis and lower extremity dysfunction using both clinician-based and patient-reported measures. Future multicenter studies with larger cohorts may help validate the present findings.

## Conclusions

In this retrospective exploratory study, patients with C3/4 stenosis demonstrated worse lower extremity-related functional impairment and were older than those without C3/4 stenosis. These findings may support the potential importance of level-specific evaluation of the upper cervical spine in CSM; however, the independent contribution of C3/4 pathology could not be definitively determined from the present analysis.

## Appendices

**Table 6:** Patient-level distribution of cervical stenosis and surgical characteristics in the C3/4 stenosis group and non-C3/4 stenosis group S group: C3/4 stenosis group; N group: non-C3/4 stenosis group Patient-level data are shown for stenosis at each cervical level, surgical procedure, decompression level, and the number of decompressed levels. Circles indicate the presence of stenosis at the corresponding cervical level

Patient no.	Age	Sex	Group	Stenosis level					Surgical procedure	Decompressed level	Number of decompressed levels
				C3/4	C4/5	C5/6	C6/7	C7/Th1			
No.1	58	M	S group	◯	◯	◯	◯	－	Skip laminoplasty	C3-7	4
No.2	77	F	S group	◯	－	－	－	－	Skip laminoplasty	C3-4	1
No.3	86	M	S group	◯	◯	◯	－	－	Skip laminoplasty	C3-7	4
No.4	77	F	S group	◯	◯	－	－	－	Skip laminoplasty	C4-6	2
No.5	75	M	S group	◯	◯	◯	◯	－	Skip laminoplasty	C3-7	4
No.6	73	M	S group	◯	－	－	－	－	Skip laminoplasty	C3-7	4
No.7	85	M	S group	◯	－	－	－	－	Skip laminoplasty	C3-4	1
No.8	72	M	S group	◯	◯	－	－	－	Skip laminoplasty	C3-6	3
No.9	88	F	S group	◯	◯	◯	－	－	Skip laminoplasty	C3-7	4
No.10	75	M	S group	◯	◯	－	－	－	Skip laminoplasty + posterior spinal fusion	C3-6	3
No.11	48	M	S group	◯	◯	◯	－	－	Skip laminoplasty	C4-7	3
No.12	51	M	N group	—	—	◯	—	—	Skip laminoplasty	C4-6	2
No.13	50	F	N group	—	—	◯	—	—	Skip laminoplasty	C4-6	2
No.14	52	M	N group	—	—	◯	◯	—	Skip laminoplasty	C4-7	3
No.15	52	M	N group	—	◯	◯	—	—	Skip laminoplasty	C4-6	2
No.16	56	M	N group	—	—	◯	—	—	Skip laminoplasty	C5-7	2
No.17	69	M	N group	—	◯	◯	—	—	Skip laminoplasty	C4-7	3
No.18	49	F	N group	—	◯	◯	◯	—	Skip laminoplasty	C4-7	3
No.19	56	M	N group	—	—	◯	—	—	Skip laminoplasty	C5-7	2
No.20	62	M	N group	—	◯	◯	◯	—	Skip laminoplasty	C3-7	4
No.21	45	M	N group	—	—	◯	—	—	Skip laminoplasty	C4-7	3
No.22	64	F	N group	—	◯	◯	—	—	Skip laminoplasty	C5-7	2
No.23	69	F	N group	—	◯	—	—	—	Skip laminoplasty + posterior spinal fusion	C4-5	1
No.24	69	M	N group	—	◯	◯	—	—	Skip laminoplasty	C4-7	3
